# Effect of Pumpkin Seed Milk on Physicochemical, Textural, Rheological, Antioxidant, and Sensory Properties of Pudding

**DOI:** 10.1002/fsn3.70806

**Published:** 2025-08-19

**Authors:** Başak Aygün, Ahmet Emirmustafaoğlu

**Affiliations:** ^1^ Department of Gastronomy and Culinary Arts Bolu Abant Izzet Baysal University Bolu Turkey

**Keywords:** dairy dessert, plant based milk, plant based milk pudding, pudding, pumpkin seed, pumpkin seed milk

## Abstract

Plant‐based milks have gained popularity in recent years due to nutritional preferences, health conditions, and other factors, but studies on products made from plant‐based milk are limited. This study aimed to increase the nutritional value, functional, and organoleptic properties of puddings produced with milk derived from pumpkin seeds. For pumpkin seed milk production, pumpkin seeds were heat treated at 90°C for 10 min by adding water at a ratio of 1:5, then crushed with a blender and filtered through a cloth. Puddings were produced by mixing pumpkin seed milk and cow's milk at ratios of 0:100 (P0), 25:75 (P25), 50:50 (P50), 75:25 (P75), and 100:0 (P100) and characterized for physicochemical, textural, rheological, antioxidant, and sensory properties. As a result of the analyses, the highest antioxidant activity, phenolic matter concentration, pH, and Δ*E* values were observed in the P100. The antioxidant activity increased from 2.38 μmol trolox/100 g in P0 to 3.79 μmol trolox/100 g in P100 (*p* < 0.05). Total phenolic matter concentration increased from 33.69 to 41.85 mg/100 g (*p* < 0.05). The use of pumpkin seed milk in pudding production caused a decrease in dry matter, ash, protein, water holding capacity, viscosity, firmness, adhesiveness, color (L*, a*, b*, C*), storage modulus (*G*′) and loss modulus (*G*″) values. The lowest *G*′ and *G*″ values were associated with P75, followed by P100. As a result of the sensory analysis, P100 had the highest color‐appearance score (8.2), while P50 and P25 received the highest scores in structure‐consistency (7.3) and taste‐odor (7.73) evaluations, respectively. P25 received the highest score in terms of general acceptability and was followed by P50. It was concluded that using up to 50% pumpkin seed milk in pudding production was sensory acceptable. The pudding's functional properties were improved and a plant‐based milk pudding was presented by utilizing pumpkin seed milk. Pumpkin seed milk can be used in milk desserts such as pudding.

## Graphical abstract

This study investigates the physicochemical, textural, rheological, antioxidant and sensory properties of puddings made with different ratios of milk obtained from pumpkin seeds. It was concluded that using up to 50% pumpkin seed milk in pudding production was sensory acceptable. Pumpkin seed milk can be used alone or in combination with cow's milk to improve the nutritional, functional and sensory properties of the pudding. Pudding made with pumpkin seed milk is an alternative product for individuals who prefer to consume plant‐based foods or who are forced to do so for health reasons.

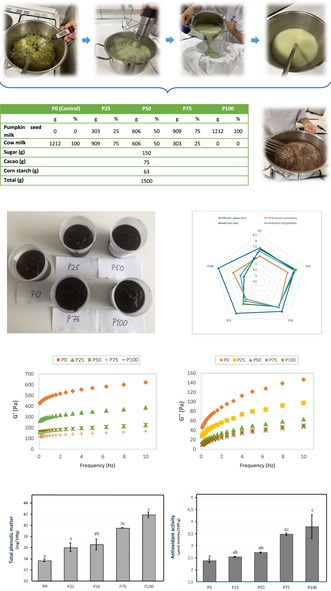

## 1 Introduction

Some people cannot consume milk and dairy products for different reasons, although regular consumption of milk and dairy products is recommended. These may be related to dietary preferences (e.g., veganism, vegetarianism), health conditions such as lactose intolerance or milk allergy, or ethical considerations. The inability to consume milk and/or the unavailability of viable milk sources has stimulated the production and marketing of plant‐based milk substitutes. These products, which are similar in appearance to cow's milk, are produced by grinding materials such as coconut, almond, hazelnut, oat, soybeans, etc., followed by extraction in water by homogenization and separation of the solid phase from the liquid phase. Cereals, pseudocereals, legumes, nuts, and seeds are used as raw materials in the production of plant‐based milks (Erk et al. 2019; Plamada et al. 2023). Plant‐based beverages are high in components such as vitamins, minerals, fiber, and antioxidants and are considered functional foods due to these properties (Arbağ 2022). Products such as lupin, peas, rice, hemp, cashew, macadamia nuts, bambara groundnut, sesame, and walnut milk are industrially produced. However, plant‐based milks derived from almonds, hazelnuts, walnuts, soy, and lupin may not be preferable for some individuals due to their allergenic potential.

Pumpkin seeds obtained by processing pumpkins of the Cucurbitaceae family contain important substances in their composition. The processing of pumpkins produces 3.1%–4.4% of seeds (Ninčević Grassino et al. 2023). Pumpkin seeds are rich in high‐quality protein and oil. Protein isolates from pumpkin seeds, which contain about 35% crude protein, are similar to those from soybeans with high bioavailability. They contain squalene, phytosterols, sucurbitacin, and phenolic compounds (Dotto and Chacha 2020; Yu et al. 2023). Pumpkin seed oils are rich in linoleic and oleic acids, vitamin E (tocopherols), and carotenoids (Luo et al. 2024). They are a good source of magnesium, manganese, phosphorus, iron, and zinc (Sarıkamış et al. 2010). In addition, proteins separated from different pumpkin seed species were shown to promote hypoglycemic effects, which indicates that they have antidiabetic properties (Syed et al. 2019). Pumpkin seed extract is effective in the prevention of prostate cancer and urinary system diseases (Yu et al. 2023). Pumpkin seeds' unsaturated fatty acids have been linked to the prevention of cardiovascular disease (Luo et al. 2024). Their available phytosterols reduce the risk of cardiovascular threats by lowering blood low‐density lipoprotein cholesterol (Dotto and Chacha 2020). They have blood pressure‐lowering, anti‐inflammatory, anticoagulant, and anti‐allergic properties. They contain antioxidant substances such as flavonoids, flavones, catechins, polyphenols, and phytosterols (Abed et al. 2024).

Pudding is a semi‐solid, gel‐like milk dessert consumed by all age groups at home or in cafes, restaurants, etc. Pudding, which can be consumed hot or cold, contains starch, sugar and/or cocoa or chocolate to milk and applying heat treatment. Increasing consumer awareness regarding dietary preferences has created different product needs in the market. For people who follow specific diets, there are so‐called “light puddings” that are sugar‐free, gluten‐free, and lower in terms of calorie content. Today, as a result of the increasing consumption of plant‐based milks, interest in products made from these milks has also increased. People prefer to use these plant‐based milks as raw materials in the production of various foods or to enrich them. To meet these consumer demands, manufacturers are including plant‐based milk products in their R&D efforts. The use of plant‐based milks also has some disadvantages. Undesirable colors, flavors, and odors originating from the raw materials used are among the main problems. The particle homogeneity of plant‐based milk is lower than that of animal milk, resulting in reduced stability. The instability of plant‐based milk can lead to floating fat and protein aggregation during storage, which affects the flavor and shelf life of milk beverages (Luo et al. 2024). Similar problems also exist with milk derived from pumpkin seeds. In a dark, sweet product such as cocoa pudding, using pumpkin seed milk is important since it reduces these unwanted flavors, colors, and aromas. There is limited research on the use of plant‐based milks in pudding. Karimidastjerd et al. (2024) formulated rice pudding with plant‐based milk alternatives such as almond, soy, oat, coconut, and pistachio milk and compared their textural, rheological, and sensory properties. Ayah et al. (2022) analyzed the physicochemical, textural, and sensory properties of puddings produced from coconut, almond, and rice milk. Karlı and Koç (2023) analyzed the total phenolic matter, antioxidant activity, and sensory properties of puddings produced with pumpkin and melon seed milk. While a comprehensive literature review was conducted, no other publication on pudding/milk dessert produced from pumpkin seed milk was identified. In this study, physicochemical, textural, rheological, antioxidant, and sensory properties of cocoa puddings produced with different percentages (25%–50%–75%–100%) of pumpkin seed milk were investigated. Thus, it was aimed to increase the nutritional value, functional and organoleptic properties of the pudding and to present an alternative product for people and producers who prefer plant‐based milk consumption and/or products with functional properties.

## 2 Materials and Methods

### 2.1 Material

Raw, unsalted, and unshelled pumpkin seeds procured from a local store were used for pumpkin seed milk production. Semi‐fat UHT cow milk (1.5% fat), cocoa, sugar, and corn starch used in pudding production were obtained from the local market.

### 2.2 Pumpkin Seed Milk Production

Three different experiments were conducted to determine the optimal water ratio for making pumpkin seed milk (pumpkin seed to water ratio of 1:1, 1:3, and 1:5). The product produced with the 1:1 dilution ratio was found to have an undesirable creamy consistency. In comparison to 1:5, the ratio of 1:3 was found to have a rougher structure, a duller color, and a particularly unpleasant pumpkin seed taste–odor. The pumpkin seed milk that was produced with a 1:5 ratio was chosen because it had better taste and odor characteristics, more brightness and vibrant appearance, and a smoother structure than the other ratios.

Water was added to the pumpkin seeds at a ratio of 1:5 (weight: weight) and heat treatment was applied at 90°C for 10 min in a stainless steel pot. After shredding with a blender (Electrolux E5HB1), it was filtered through a cloth to avoid leaving a granular structure in the mouth, and the resulting filtrate was used as pumpkin seed milk.

### 2.3 Pudding Production

Puddings were produced using the cocoa pudding recipe by İspirli et al. (2018). Pudding was produced in two duplicates using all the ingredients required in the amounts listed in Table 1. First, milk, cocoa, and corn starch were placed in a saucepan and heated for 15 min. Following this step, sugar was added, and heat was maintained for another 5 min. To achieve a homogeneous and smooth structure, the mixture was continuously stirred with a whisk throughout the heat treatment. The puddings were placed in plastic containers and left at room temperature to cool before being sealed and stored at +4°C for analysis. All analyses except texture and sensory analyses were carried out by taking sufficient samples from these containers. Since transferring pudding from one container to another would cause a change in its textural and sensory properties, the samples required for texture and sensory analyses were placed in different containers with lids during production and stored at +4°C, and the analyses were carried out in these containers.

**TABLE 1:** Pudding formulation.

	P0 (Control)		P25		P50		P75		P100	
	g	%	g	%	g	%	g	%	g	%
Pumpkin seed milk	0	0	303	25	606	50	909	75	1212	100
Cow milk	1212	100	909	75	606	50	303	25	0	0
Sugar (g)	150									
Cacao (g)	75									
Corn starch (g)	63									
Total (g)	1500									

### 2.4 Physicochemical Analysis

Dry matter and mineral matter were determined by gravimetric method, and protein was determined by Kjeldahl method (Metin and Öztürk 2017). pH was measured using a pH meter (WTW İnolab pH 720).

### 2.5 Color Analysis

L*, a* and b* values of the samples were measured with a color analyzer (Minolta, CR400, Japan). L* stands for lightness, a* for red‐greenness, and b* for yellow‐blueness. Also, to reveal the color difference between the control pudding P0 and the pudding samples produced using pumpkin seed milk (P25, P50, P75, and P100), the C* value and delta *E* (Δ*E*) value were calculated using the following Equations (1) and (2) (Aydemir and Kurt 2020; Chudy et al. 2019).

(1) $$C^{*}=\sqrt{a^{*2}+b^{*2}}$$

(2) $$\Delta E=\sqrt{{\left(L1-L2\right)}^{2}+{\left(\;a1-a2\right)}^{2}+{\left(b1-b2\right)}^{2}}$$

### 2.6 Water Holding Capacity (WHC), Viscosity and Texture Analyses

WHC was determined using the method described by Granato et al. (2010). A sample weighing 10 g (DS) was centrifuged at 5000 rpm at 20°C for 40 min (Sigma, 2‐16 K, Germany). After the liquid portion (DE) was removed from the sample, the centrifuge tube was weighed. WHC was calculated using the following formula Equation (3):

(3) $$\mathrm{WHC}\;\left(\%\right)=\left[\left(\mathrm{DS}-\mathrm{DE}\right)/\mathrm{DS}\right]\times 100$$

A viscometer (AND Viscometer SV‐10, Japan) was used to determine the viscosity of the samples. The temperature of the samples was brought to 10°C ± 2°C before analysis. The average of the results collected every 15 s over 1 min was used.

The textural parameters of the pudding samples were determined with a texture analyzer (Stable Microsystems, Surrey, UK). Pudding samples were stored in cylindrical containers at +4°C for 1 day following production. The temperature of the samples was brought to 10°C ± 1°C before analysis. The samples were analyzed using a P/36R cylindrical probe (P/36R; 36 mm dia aluminum radiused AACC) at a test speed of 1 mm/s and a test duration of 5 s. The cylindrical probe was immersed 12 mm into the sample container and returned to the starting point. Firmness and adhesiveness values were examined in the samples. Firmness was recorded as the height of the force peak during compression, and adhesiveness was recorded as the negative force area during compression (Arltoft et al. 2008; Sagdic and Emirmustafaoglu 2025).

### 2.7 Rheological Analysis

The rheological properties of the samples were determined by the method described by Zheng et al. (2017) using the Kinexus Pro rheometer (Malvern Instruments Ltd., UK). The rheological test was performed at 10°C using a 4 cm diameter parallel plate probe with a 0.5 cm gap size. The pudding sample was placed on the bottom plate; then the top plate was slowly moved downwards, and the excess was scraped off from the plate. Dynamic viscoelasticity screening test was performed at 10°C with 1% screening strain. Storage modulus (*G*′), viscous modulus (*G*″) and loss angle tangent (tanδ = *G*″/*G*′) values were recorded in the angular frequency range of 0.1–10 Hz.

### 2.8 Determination of Total Phenolic Content and Antioxidant Activity

The extract required for total phenolic content and antioxidant activity analyses was obtained by modifying the method reported by Thaipong et al. (2006). 3 g of sample was homogenized with 25 mL of pure methanol at 21,000 rpm for 2 min (MICCRA D‐9 Homogenizer, MICCRA GmbH, Müllheim, Deutschland). After this process, the mixture was kept at +4°C overnight and then centrifuged at 10,000 rpm for 20 min. The supernatant phase was removed and completed with methanol in a 25 mL volumetric flask. The extract obtained in this way was stored at −20°C until used for analysis.

The total phenolic content of the samples was determined using the Folin–Ciocalteu method (Singleton and Rossi 1965). 7 mL of pure water was added to 500 μL of diluted pudding extract. After mixing 500 μL of Folin–Ciocalteu solution with the prepared pure water and pudding extract, 2 mL of saturated sodium carbonate was added and left in the incubator at 25°C for 1 h. The blue color formed by the samples after the reaction was read against air at 720 nm wavelength with a spectrophotometer (UV 1800, Shimadzu, Japan). The total phenolic amount was determined according to the reference standard gallic acid curve and calculated using the linear equation of the standard curve with the formula given in Equations (4) and (5):

(4) y=0.0066x−0.0254

(5) $$R^{2}=0.9968$$

The antioxidant activity of the samples was determined according to the TEAC (Trolox Equivalent Antioxidant Activity) method (Re et al. 1999). ABTS* + (2,2′‐azinobis‐3‐ethylbenzothiazoline‐6‐sulfonic acid) radical solution and ethanol mixture were placed into the spectro cuvette, and the absorbance of the mixture was fixed between 0.720 and 0.680. After adding 5 μL of sample extract to 1 mL of ABTS* + radical solution in the spectro cuvette, the absorbance value was measured at 734 nm wavelength for 6 min. The absorbance measured at the end of the period was taken as the basis, and the percentage inhibition rate was calculated according to the value measured at the beginning according to the formula Equation (6):

(6) $$\text{Inhibition rate}\;\left(\%\right)=\frac{\text{Initial absorbance value}-\text{Final absorbance value}}{\text{Initial absorbance value}}\times 100$$

The antioxidant activity of the samples was determined according to the Trolox standard curve, and the percentage inhibition values of the ABTS* + radical were calculated by preparing Trolox solutions of different concentrations.

### 2.9 Sensory Analysis

The color‐appearance, structure‐texture, taste‐odor, and overall acceptability of puddings produced with cow's milk and pumpkin seed milk were evaluated by scoring test. Sensory analyses were performed with 15 panelists between the ages of 22 and 46, including faculty members and graduate students of the Food Engineering and Gastronomy and Culinary Arts departments of Bolu Abant İzzet Baysal University. Sensory evaluations were performed in a room with white light and natural ventilation. Samples were presented in 30 cc transparent plastic containers at 10°C with three‐digit codes, and the panelists were given water to rinse their mouths between each pudding sample. The panelists used a 9‐point hedonic scale (1 = Inedible, 5 = Neither good nor bad, 9 = Excellent) to evaluate the five different pudding samples. The study was approved by Bolu Abant İzzet Baysal University Ethics Committee with registration number 2022/424.

### 2.10 Statistical Analysis

All the analyses were repeated twice; the resulting data were subjected to analysis of variance (ANOVA). Duncan post hoc test was used to differentiate the means between the groups at the *p* < 0.05 level. SPSS (IBM Statistics 24, USA) program was used for statistical analysis.

## 3 Results and Discussion

### 3.1 Physicochemical Properties

The physicochemical analysis results of pudding samples made with pumpkin seed milk and cow milk are given in Table 2.

**TABLE 2:** Physicochemical properties of puddings.

	Pudding samples (X̄±SD)				
	P0	P25	P50	P75	P100
Dry matter (%)	29.19 ± 0.449^a^	27.81 ± 0.146^b^	26.31 ± 0.298^c^	24.84 ± 0.434^d^	23.43 ± 0.375^e^
Ash (%)	1.00 ± 0.025^a^	0.93 ± 0.020^b^	0.83 ± 0.011^c^	0.76 ± 0.014^d^	0.70 ± 0.020^e^
Protein (%)	4.41 ± 0.025^a^	3.92 ± 0.043^b^	3.40 ± 0.101^c^	2.98 ± 0.073^d^	2.24 ± 0.293^e^
pH	7.28 ± 0.028^a^	7.43 ± 0.028^b^	7.65 ± 0.032^c^	7.81 ± 0.032^d^	7.91 ± 0.014^e^

*Note:* Means with different letters are statistically different (*p* < 0.05).

Abbreviations: SD, standard deviation; X̄, mean.

The dry matter values of puddings produced using pumpkin seed milk were found to be lower than those of cow's milk pudding. The results indicate that the P0 sample has the highest dry matter value at 29.19% and the P100 sample has the lowest at 23.43%. Dry matter decreased as the amount of pumpkin seed milk used in pudding production increased (*p* < 0.05). Because pumpkin seeds have a high dietary fiber content, increasing the proportion of pumpkin seed milk used in pudding production increased moisture content, resulting in a decrease in dry matter. It has been reported that both soluble and insoluble dietary fiber components bind to water, increasing moisture content (Ng et al. 2023). The addition of water to the production of pumpkin seed milk reduced the dry matter value of the milk and the dry matter values of the puddings made from it. The decrease in protein and ash values (Table 2), correlated to the use of pumpkin seed milk, resulted in lower dry matter values. The fact that the dry matter value of pumpkin seed milk used in pudding production is lower than semi‐fat cow's milk has been effective in obtaining this result. Kuru and Tontul (2020) reported that the dry matter value of pumpkin seed milk obtained by diluting 1:5 ranges between 7.7% and 9.3%. Sucak et al. (2020) reported that the average dry matter content of five trademarked low‐fat UHT cow's milk products in Turkey was 9.98%. Saleh (2022) reported a dry matter content of 37.65% in the control group of cocoa puddings produced from cow's milk using 6% starch and 25% sugar, higher than our study results. This result was achieved because the 5.19% starch and 12.37% sugar ratio used in our study was lower and the heat treatment time was different. Similar to the present study, Karimidastjerd et al. (2024) reported that rice pudding made with plant milks—such as almond, soy, oat, coconut, and pistachio—had lower dry matter values than rice pudding made with cow's milk. Jasper et al. (2020) reported that the moisture content of breads enriched with pumpkin seed milk increased with an increase in the proportion of pumpkin seed milk in the formulation.

The use of pumpkin seed milk decreased the ash value of the pudding (*p* < 0.05). The ash level of the pudding samples significantly decreased (*p* < 0.05) as the amount of pumpkin seed milk increased. The ash value, which was 1% in P0, decreased to 0.70% in P100. Because cow's milk contains more ash than pumpkin seed milk, puddings made using pumpkin seed milk have lower ash levels. Jeske et al. (2017) reported that cow's milk has higher ash values than plant‐based milks (except soy milk). Ash is the inorganic portion of food, consisting of minerals and trace elements such as calcium and magnesium. Because cow's milk is rich in micronutrients and minerals such as calcium and iodine (Ng et al. 2023), puddings formulated with higher proportions of cow's milk have been found to have higher ash values. The findings are consistent with the dry matter values. Pumpkin seed has a rich mineral composition and is a good source of magnesium, potassium, phosphorus, zinc, manganese, iron, calcium, sodium, and copper (Dotto and Chacha 2020). The addition of water to the production of pumpkin seed milk reduced the amount of rich ash, and as a result, the ash content of pumpkin seed milk puddings decreased. While shelled pumpkin seeds have an ash value of 3.02% (Hassan et al. 2012), the ash value of pumpkin seed milk is 0.23%–0.34% (Kuru and Tontul 2020). Seçim and Uçar (2014) determined the ash content of cocoa puddings produced from cow's milk as 0.66%–0.77%. This value is lower than the control group in our study. The difference has resulted from variations in the proportions of the ingredients used to make the pudding, as well as the intensity and duration of the heat treatment. Several studies showed products made using plant‐based milk had lower ash values than those made with cow milk. Karimidastjerd et al. (2024) reported that the ash content of rice puddings made from different plant milks was statistically significantly lower than that of rice pudding made from cow milk. Atalar (2019) reported that kefirs produced from hazelnut milk have lower ash than cow milk kefir and that ash values decrease significantly with the increase in the use of hazelnut milk in kefir production (*p* < 0.05). Rahim et al. (2022) reported that the ash value of soy milk puddings was lower than that of cow milk pudding. The results obtained in these studies are consistent with our results.

The puddings produced with pumpkin seed milk had a lower protein value than the puddings made with cow's milk (P0). Increasing the ratio of pumpkin seed milk used reduced the protein values of the puddings (*p* < 0.05), and the protein value, which was 4.41% in the control (P0), decreased to 2.24% in P100. The decrease in protein content is due to pumpkin seed milk containing less protein than cow's milk. The manufacturer reports the protein content of the semi‐fat cow's milk used in this study as 3%. Sucak et al. (2020) found the average protein value of 5 commercially sold semi‐fat UHT cow's milk products that are available in the Turkish market as 2.73%. Pumpkin seed milk has a lower protein level than cow milk due to the addition of water during production. The protein content of pumpkin seed milk, which is made from pumpkin seeds that contain 33.44% protein (Hassan et al. 2012), ranges between 0.17 and 0.34 g/100 mL (Kuru and Tontul 2020). Consistent with our findings, Plamada et al. (2023) and Jeske et al. (2017) reported that plant‐based milks have lower protein values than cow's milk, and only soy‐based milks have protein ratios close to cow's milk. Furthermore, animal proteins are more digestible and nutritious than plant proteins. Karimidastjerd et al. (2024) reported that protein values decreased when vegetable milks are used in rice pudding production. Görgün (2022) reported that protein values decreased with the use of chestnut milk in kefir production. Asaduzzaman et al. (2025) found that the protein content of noodles decreased with the addition of pumpkin seeds. These results are compatible with the findings of the present study.

The use of pumpkin seed milk in pudding production increased pH values. The pH value, which was 7.28 in cow milk pudding, increased to 7.91 in pumpkin seed milk pudding (*p* < 0.05). The use of alkalized cocoa in pudding production has led to pH levels above 7. Since the pH of pumpkin seeds (6.91) (Navarro‐Cortez et al. 2016) is higher than that of cow's milk (6.54–6.65) (Ajmal et al. 2019), the pH of the pudding samples increased as the use of pumpkin seed milk increased. Moreover, the presence of lactose and organic acids in the milk causes cow's milk pudding to become more acidic. Öztürkoğlu Budak et al. (2016) reported that pH increased with the addition of different nuts in yogurt production; this could be due to dietary fiber and proteins from nuts. The dietary fiber and protein in the pumpkin seeds used in our study are thought to have a similar effect. In line with our research, Hassan et al. (2012) found that using pumpkin seed milk to make cereal‐based probiotic beverages significantly increased pH (*p* < 0.05), while Auti (2024) found that using pumpkin seed powder in milkshakes made a significant increase in pH (*p* < 0.05). Dabija et al. (2018) reported that the addition of pumpkin seeds to yogurt increased the pH. In another study, it was stated that the pH values of plant‐based milks are close to the pH value of cow's milk (6.64) and approach neutral pH (Reyes‐Jurado et al. 2023). Atalar (2019) reported that pH values increased with the increase in the hazelnut milk ratio used in kefir production. Irkin and Güldaş (2011), İspirli et al. (2018), and Silkin et al. (2023) reported the pH value of the control group cocoa puddings produced from cow's milk as around 7. The results obtained in these studies are consistent with our findings.

### 3.2 Color Properties

Table 3 presents the pudding sample color analysis results.

**TABLE 3:** Color properties of puddings.

	Pudding samples (X̄±SD)				
	P0	P25	P50	P75	P100
L*	27.34 ± 0.018^a^	25.72 ± 0.180^b^	24.95 ± 0.216^c^	24.25 ± 0.272^d^	23.62 ± 0.230^e^
a*	7.00 ± 0.035^a^	6.85 ± 0.078^ab^	6.69 ± 0.085^b^	6.11 ± 0.081^c^	5.87 ± 0.074^d^
b*	7.11 ± 0.053^a^	7.02 ± 0.180^a^	6.61 ± 0.258^b^	6.05 ± 0.021^c^	5.85 ± 0.138^c^
C*	9.97 ± 0.064^a^	9.81 ± 0.180^a^	9.40 ± 0.244^b^	8.59 ± 0.046^c^	8.29 ± 0.148^c^
Δ*E*	—	1.64 ± 0.219^a^	2.47 ± 0.276^b^	3.39 ± 0.226^c^	4.09 ± 0.255^d^

*Note:* Means with different letters are statistically different (*p* < 0.05).

Abbreviations: C*, chroma; SD, standard deviation; X̄, mean; Δ*E*, delta *E*.

The Maillard reaction—a non‐enzymatic browning reaction—causes color changes in foods containing protein and sugar, such as pudding. Although the same amounts of ingredients were used in all pudding samples and the same production parameters were applied, the lower protein content of pumpkin seed milk led to less browning due to reduced protein/sugar interaction in the Maillard reaction. This situation affected all color parameters examined.

The L* values of the pudding samples ranged from 27.34 (at P0) to 23.62 (at P100) (Table 3). It was determined that L* values decreased as the amount of pumpkin seed milk used increased (*p* < 0.05). In other words, puddings made with pumpkin seed milk had a lower brightness than cow milk pudding, and the brightness fell as the pumpkin seed milk ratio in the formulation increased. Differences in particle size of samples containing pumpkin seed milk caused differences in light scattering and affected the L* value. The presence of greenish color in milk obtained from pumpkin seeds due to pigments such as chlorophyll has caused the L* value of puddings produced using pumpkin seed milk to decrease. On the other hand, the white color of cow's milk caused the pudding made from cow's milk to have a higher L* value. Because pumpkin seed milk has a lower L* value than cow's milk, pumpkin seed milk pudding has a lower L* value. L* values of pumpkin seed milk and low‐fat UHT cow's milk were reported as 73.78 (Luo et al. 2024) and 93.78 (Urgu et al. 2017), respectively. According to Jeske et al. (2017), plant‐based milks showed significantly less whiteness index values than cow milk. Consistent with our research, Dabija et al. (2018) and (Yeniçeri 2019) reported that L* values decreased with the addition of pumpkin seeds to yogurt, Raina et al. (2023) with the addition of pumpkin seed flour to pasta, Karaś et al. (2024) with the addition of pumpkin seed flour to wafers, Asaduzzaman et al. (2025) with the addition of pumpkin seed to cookies, and Costa et al. (2018) with the use of pumpkin seed flour in panbread formulations. Karimidastjerd et al. (2024) found that using plant‐based milks to make rice pudding significantly decreased the L* value (*p* < 0.05).

The a* values decreased with the use of pumpkin seed milk in pudding production. The a* value, which was 7.00 at P0, decreased to 5.87 at P100 (*p* < 0.05). As the percentage of pumpkin seed milk in the pudding formulation increased, the a* values decreased. This was caused by the fact that pumpkin seed milk's a* value (−2.79; Luo et al. 2024) was lower than that of cow's milk (−0.23; Urgu et al. 2017). Pigments such as chlorophyll in pumpkin seeds cause the pumpkin seed to be green. These pigments were released into the environment and increased the greenness during the heat treatments used to produce milk from pumpkin seeds and to make pudding. So pumpkin seed milk actually has a negative a* value. Cow milk is slightly green due to the presence of coloring compounds such as riboflavin, but not as much as pumpkin seed milk. In this study, the use of cocoa in pudding production caused the a* values of the samples to be positive due to the redness it created. Our findings are similar to those of Costa et al. (2018), who found that the a* value decreased in the pan bread recipe made using shelled pumpkin seed flour; Karaś et al. (2024), who reported that the a* value of wafers decreased with the addition of pumpkin seeds; Asaduzzaman et al. (2025), who reported that the a* value of cookies decreased with the addition of pumpkin seeds; and Yeniçeri (2019), who reported that the a* value in yogurt decreased significantly (*p* < 0.01) with the addition of pumpkin seeds.

Among the pudding samples, the highest b* value was measured at P0 (7.11) and the lowest value was measured at P100 (5.85) (*p* < 0.05). The use of pumpkin seed milk decreased b* values in pudding. The b* value of semi‐fat UHT cow's milk (11.07) being higher than the b* value of pumpkin seed milk (6.98) was effective in obtaining this result (Urgu et al. 2017; Luo et al. 2024). Although pumpkin seeds are rich in carotenoids that give yellowness to foods (Kaur and Sharma 2018), the water added in the production of pumpkin seed milk caused the yellowness (b* value) to decrease in the final product. Because carotenoids are insoluble in water, slightly soluble in vegetable oils, and fairly soluble in hydrocarbons such as chloroform (Ötleş and Atlı 1997). Şenyurt and Yangılar (2024) used different levels of pumpkin seed flour to make vegan amaranth milk. They found a decrease in b* values as the ratio of pumpkin seed flour they used increased. Dabija et al. (2018) found lower b* values in yogurts with added pumpkin seeds compared to their control group. Karaś et al. (2024) and Asaduzzaman et al. (2025) reported similar effects on wafers and cookies, respectively, with the addition of pumpkin seeds. These results are compatible with the present study.

The C* value, which is also referred to as chroma, is a parameter related to the color saturation in food analysis (i.e., higher C* values indicate that the color is vivid, while lower C* values indicate that the color is dull). The use of pumpkin seed milk decreased the C* value of the pudding. The use of pumpkin seed milk above 25% caused a significant decrease in C* values (*p* < 0.05). As the amount of pumpkin seed milk in the formulation increased, the C* values of the pudding samples decreased. It was determined that the pudding produced only from cow's milk (P0) was the brightest sample with the highest C* value, while the pudding made from 100% pumpkin seed milk (P100) was the dullest sample with the lowest C* value. Jeske et al. (2017) reported that plant‐based milks had statistically significantly lower whiteness index values than cow's milk. Akalın et al. (2024) attributed the difference in color parameters of dessert samples produced from plant‐based milk to carotenoids and flavonoids, which are responsible for the red, yellow, and orange colors in dairy and plant‐based milk.

According to the Δ*E* results calculated to measure the color difference between the control (P0) and the other groups, the highest value was in P100 (4.09) and the lowest was in P25 (1.64) (*p* < 0.05). It was determined that as the ratio of pumpkin seed milk used in pudding production increased, the Δ*E* value increased (*p* < 0.05). The difference in color is considered very significant for Δ*E* > 3, significant for 1.5 < Δ*E* < 3, and minor for 1.5 < Δ*E* (Pathare et al. 2013). Accordingly, the color difference can be classified as very significant for P75 and P100 samples and significant for P25 and P50. So, P25 and P50 are more acceptable in terms of color. Pigments such as chlorophyll and carotenoids in the composition of pumpkin seeds caused this difference. Reyes‐Jurado et al. (2023) reported that differences in color values with the use of plant milk may be linked to the levels of molecules responsible for color, called chromophores, and the concentration of particles present.

### 3.3 Water Holding Capacity (WHC), Viscosity and Textural Properties

In semi‐solid foods such as pudding, syneresis (water loss) occurs as a result of increased molecular relationships between amylose and amylopectin components through amylose retrogradation (Lin et al. 2025). In pudding gel, it is desired that water separation from the structure is very low; in other words, that the WHC is high. The WHC values of the pudding samples decreased with the use of pumpkin seed milk (Table 4). The WHC value, which was 81.21% at P0, decreased to 72.52% at P100. Only the WHC values of P75 and P100 samples were statistically different from the control (*p* < 0.05). Increased pumpkin milk usage resulted in lower WHC values. The protein and fiber in pumpkin seeds give it a certain water retention capacity. As seen in Table 2, the decrease in protein and dry matter content with the use of pumpkin seed milk in pudding production was effective in decreasing WHC values. Milk proteins increase gel strength, while increasing concentrations of starch reduce syneresis. Inclusion of water‐binding ingredients such as hydrocolloids or sugar effectively reduces syneresis in starch‐based gels (Lin et al. 2025). The use of water in the production of pumpkin seed milk resulted in a decrease in protein and fiber content, resulting in a decrease in the WHC of the pudding. In addition, variations in some protein characteristics of proteins in cow's milk and pumpkin seed milk were effective in obtaining different WHC values in pudding samples. The amino acid composition, structure, and the surface polarity/hydrophobicity ratio of proteins affect the water holding capacity of foods (Atalar 2019). In a study, it was reported that yogurts produced with the addition of hazelnuts, almonds, and walnuts had lower WHC values than the control group produced with only cow's milk, consistent with our study (Öztürkoğlu Budak et al. 2016). It was also reported that the use of chestnut milk up to 40% reduced the WHC values of kefir samples (Görgün 2022).

**TABLE 4:** WHC, viscosity, and textural properties of puddings.

	Pudding samples (X̄±SD)				
	P0	P25	P50	P75	P100
WHC (%)	81.21 ± 0.159^a^	77.51 ± 4.47^ab^	76.05 ± 5.632^ab^	73.36 ± 6.697^b^	72.52 ± 0.202^b^
Viscosity (mPa.sn)	1.49 ± 0.099^a^	1.11 ± 0.499^b^	0.95 ± 0.163^b^	0.82 ± 0.244^b^	0.81 ± 0.035^b^
Firmness (g)	132.64 ± 4.510^a^	97.74 ± 27.858^ab^	90.80 ± 15.919^ab^	75.60 ± 19.898^b^	85.55 ± 1.061^b^
Adhesiveness (g.sec)	600.79 ± 27.451^a^	383.21 ± 140.898^a^	349.98 ± 147.347^a^	320.37 ± 176.312^a^	290.89 ± 102.341^a^

*Note:* Means with different letters are statistically different (*p* < 0.05).

Abbreviations: SD, standard deviation; WHC, water holding capacity; X̄, mean.

The viscosity of the puddings produced using pumpkin seed milk was lower than that of the pudding made from cow's milk (*p* < 0.05). Viscosity values were shown to decrease as the amount of pumpkin seed milk increased. When compared to cow's milk, pumpkin seed milk has lower dry matter, protein, and WHC values, which result in puddings with a lower viscosity value. The high viscosity value of the substance is due to the swelling of starch granules as a result of water absorption. Consistent with the results, Hassan et al. (2012) reported that the use of 10% pumpkin seed milk in the production of cereal‐based probiotic beverages significantly decreased viscosity (*p* < 0.05). Yeniçeri (2019) reported that adding pumpkin seeds to yogurt caused a significant (*p* < 0.01) decrease in viscosity values compared to the control sample. Rahim et al. (2022) reported that the viscosity of the pudding decreased with the addition of soy milk compared to the control. Also, Ayah et al. (2022) found that puddings produced from coconut, almond, and rice milk had lower viscosity than those produced from cow's milk.

The firmness values of the pudding samples also decreased with the use of pumpkin seed milk. The lowest firmness value was observed for sample P100 as 85.55 g, while the highest value was observed for sample P0 as 132.64 g. The firmness values decreased as the amount of pumpkin seed milk in the formulation increased. Only the firmness values of P75 and P100 samples were different from the control (*p* < 0.05). In other words, using up to 50% pumpkin seed milk did not cause a significant difference in the firmness values of the pudding. This is important for consumer acceptance. Puddings have a typical semi‐solid texture resulting from the interaction of milk proteins with starch or other hydrocolloids (Karimidastjerd et al. 2024). Although the same amount of starch was used as a thickener in the production of the pudding samples, the P0 sample had greater firmness than the others since it retained more water due to its relatively dry matter and protein contents. Similarly, Karimidastjerd et al. (2024) reported that puddings made with plant‐based milk had lower hardness values than puddings made from cow's milk. Ayah et al. (2022) reported that the hardness value of the pudding produced from almond milk was lower than that produced from cow's milk, while those made from rice and coconut milk were higher than that of cow's milk. It was reported that the hardness values decreased with the addition of pumpkin seeds to the wafer (Karaś et al. 2024). These findings are consistent with our research.

The use of pumpkin seed milk reduced the adhesiveness value of the pudding. As the percentage of pumpkin milk in the pudding formulation increased, the adhesiveness values decreased. P100 has the lowest adhesiveness value (290.89 g.sec), while P0 has the highest (600.79 g.sec). The increased adhesiveness of pudding produced from cow's milk can be attributed to the increased water in the gel due to decreased syneresis (Dabija et al. 2018). Our result is consistent with the study by Abdo Qasem et al. (2017), in which they reported that the adhesiveness values of the pudding samples produced by adding seedless okra shells were significantly lower than the control (*p* < 0.05).

### 3.4 Rheological Analysis Results

**FIGURE 1:**
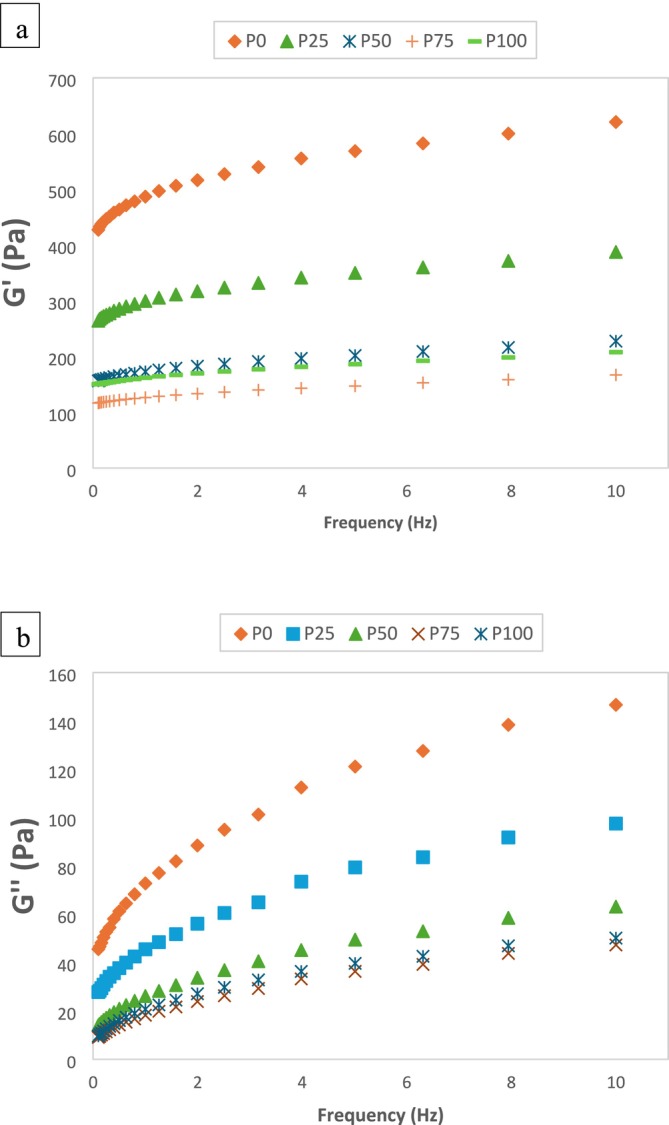
(a) Storage modulus (*G*′) (b) Viscous modulus (*G*″) of pudding samples.

Storage modulus (*G*′) and loss modulus (*G*″) values rose with increasing frequency in all pudding samples made from pumpkin seed milk and/or cow milk (Figure 1a,b). Since the *G*′ value is higher than the *G*′ value at each frequency and there is no phase transition between *G*′ and *G*″, the structure of the pudding samples is a viscoelastic solid. The use of pumpkin seed milk decreased both *G*′ and *G*″ values of the pudding samples. P75 has the lowest *G*′ and *G*″ values, followed by P100. A higher *G*′ value indicates stronger interactions between particles, while a lower *G*′ value indicates weaker bonds within the matrix (Karimidastjerd et al. 2024). It can be deduced that P75 and P100 have weaker viscoelasticity than the other samples. The decrease in dry matter, protein, and WHC values of puddings with the use of pumpkin seed milk (Tables 2 and 4) was effective in the *G*′ and *G*″ values being lower than cow milk pudding. Karimidastjerd et al. (2024) found that puddings produced from plant‐based milks, except for soy‐milk, have lower G' and G" values than puddings produced from 1.5% fat cow's milk. The rheological properties of pudding, which is generally a milk protein‐based starch paste, are between gel and liquid (Lim and Narsimhan 2006). Alamprese and Mariotti (2011) reported that puddings exhibited a solid‐like behavior typical of semisolid foods, with *G*′ being higher than *G*″ at all frequencies. Similarly, Zheng et al. (2017) reported that the *G*′ value was higher than the *G*″ in milk pudding samples.

**FIGURE 2:**
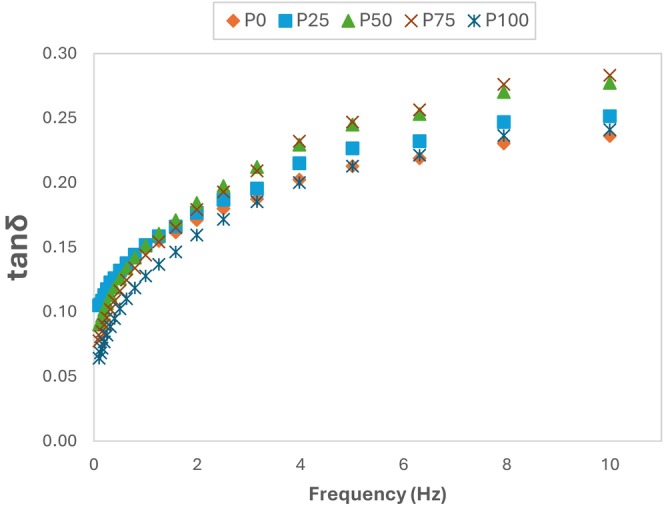
Loss angle tangent (tanδ) of pudding samples.

Viscoelasticity is expressed by loss tangent (tanδ), which is the ratio between the *G*″ and the *G*′. While a lower tanδ implies a more elastic composition and the characteristics of a solid, a higher tanδ indicates a highly viscous composition (Zheng et al. 2017). We determined that the pudding samples' tanδ values ranged from 0.06 to 0.28 (Figure 2). The highest tanδ was found for P75 and the smallest for P0. Tanδ< 1 indicates that the sample has viscoelastic character. Therefore, our samples have viscoelastic character. Karimidastjerd et al. (2024) found the tanδ value to be < 1 in rice puddings produced with different plant‐based milks and cow's milk, and reported that all pudding samples showed a soft gel behavior.

### 3.5 Total Phenolic Matter and Antioxidant Activity Values

**FIGURE 3:**
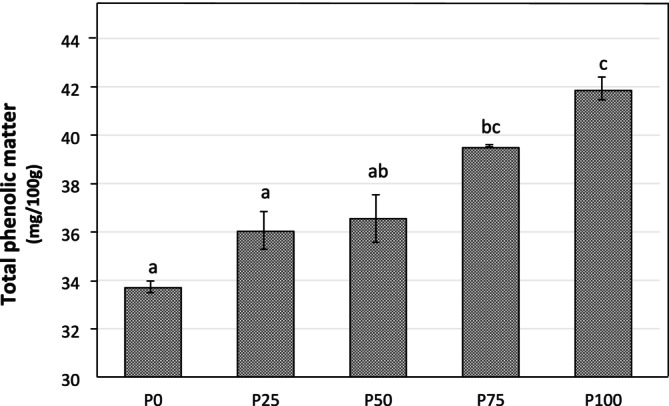
Total phenolic content of pudding samples.

As illustrated in Figure 3, the total phenolic content, which was 33.69 mg/100 g in cow's milk pudding, increased to 41.85 mg/100 g in 100% pumpkin seed milk pudding (*p* < 0.05). All pudding samples using different proportions of pumpkin seed milk had higher total phenolic matter than the control group. The increasing ratio of pumpkin seed milk increased the total phenolic matter. This outcome was due to the high phenolic content of pumpkin seeds and the milk made from them. The most common phenolics found in pumpkin are chlorogenic acid, quercetin, caffeic acid, gallic acid, p‐coumaric acid, and ferulic acid (Yang et al. 2022). Kuru and Tontul (2020) determined the total phenolic content of pumpkin seed milk to be between 212.6 and 423.2 mg GAE/L (mg gallic acid equivalents). Karlı and Koç (2023) reported the total phenolic content of pumpkin seed milk as 442.21 mg GAE/100 g, and the total phenolic content of the pudding made from this milk as 398.04 mg GAE/100 g. The value of 37.63 mg GAE/100 g in pudding made from cow's milk is consistent with the findings of the present study. The lower total phenolic substance value of our pumpkin seed milk puddings compared to Karlı and Koç (2023) may be due to the water ratio used in pumpkin seed milk production and the degree and duration of heat treatment applied. Depending on consumer preferences, puddings can be produced with more viscous or fluid characteristics. Applying heat treatment for longer periods or at higher temperatures to obtain more viscous products leads to the loss of phenolic substances. In another study, it was reported that the use of pumpkin seed flour in vegan amaranth milk production increased phenolic substance values (Şenyurt and Yangılar 2024). Raina et al. (2023) stated that the phenolic content increased with the addition of pumpkin seed flour to pasta. Karaś et al. (2024) reported that the addition of pumpkin seed flour increased the phenolic content of wafers. Asaduzzaman et al. (2025) also found that the phenolic compound of cookies and noodles increased with the addition of pumpkin seeds. These results are consistent with our study.

**FIGURE 4:**
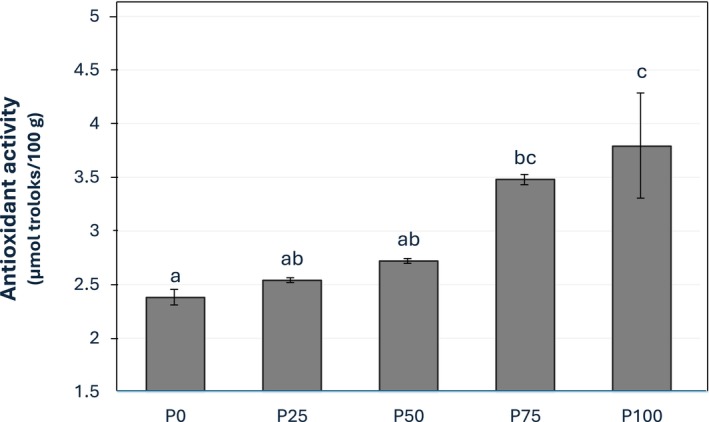
Antioxidant activity of puddings.

Pumpkin seed milk pudding has higher antioxidant activity values than cow milk pudding (Figure 4). The antioxidant activity values of the pudding samples increased in line with the amount of pumpkin seed milk used in production. The antioxidant activity values of P75 (3.48 μmol/100 g) and P100 (3.79 μmol/100 g) were significantly higher than that of P0 (2.38 μmol/100 g) (*p* < 0.05). Plant phenolics generally have high antioxidant capacity. Therefore, the increase in antioxidant activity value in relation to the usage of pumpkin seeds is an expected outcome. Pumpkin seeds, despite their small size, are extremely high in health‐promoting nutraceuticals (Fatima et al. 2025). It has been reported that antioxidant activity increases when products containing plant phenols are added to different foods. Atalar (2019) found that antioxidant capacity increased with the increasing hazelnut milk ratio used in kefir formulation. In addition, some antioxidant compounds are formed during the Maillard reaction seen in foods containing protein and carbohydrates, such as pudding. These include the production of polymers with antioxidant properties and the transformation of Amadori compounds into amino reductones. Some heterocyclic compounds also show antioxidant activity. For instance, the antioxidant activity of pyrrole is quite high (Sun et al. 2007). Karlı and Koç (2023) reported that pumpkin seed milk pudding showed higher percent inhibition of DPPH (1,1‐difenil‐2‐pikrilhidrazil) radical than cow milk pudding. Şenyurt and Yangılar (2024) noticed that the sample with the highest amount of pumpkin seed flour had the highest antioxidant activity, and that amaranth milk made with pumpkin seed flour had higher antioxidant activity than the control. In another study, it was reported that the antioxidant activity of pasta showed an increase with the addition of pumpkin seed flour (Raina et al. 2023). According to Karaś et al. (2024), adding pumpkin seed flour to wafers increased their antioxidant activity. Similar results were reported by Gebremariam et al. (2024) in cookie samples. The results obtained in these studies are consistent with the results reported in this study.

### 3.6 Sensory Properties

**FIGURE 5:**
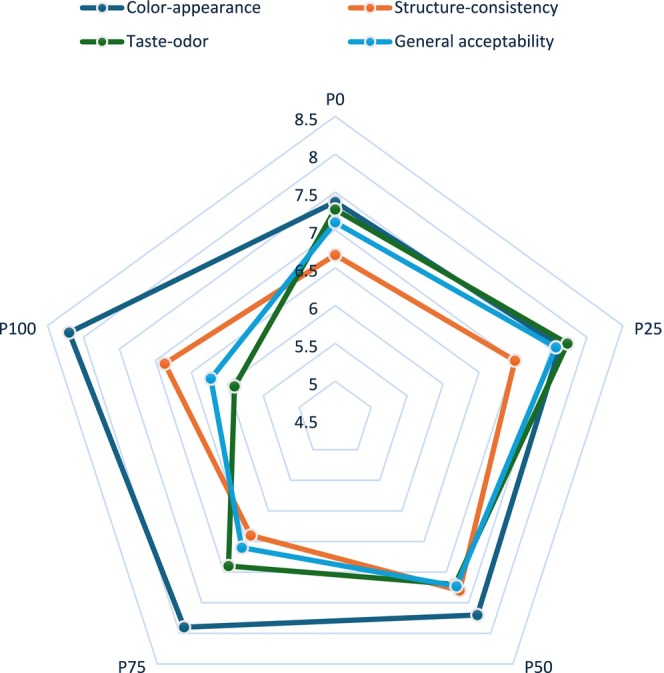
Sensory evaluation results of pudding.

The sensory analysis results of the pudding samples made with pumpkin seed milk and cow's milk are given in Figure 5.

**FIGURE 6:**
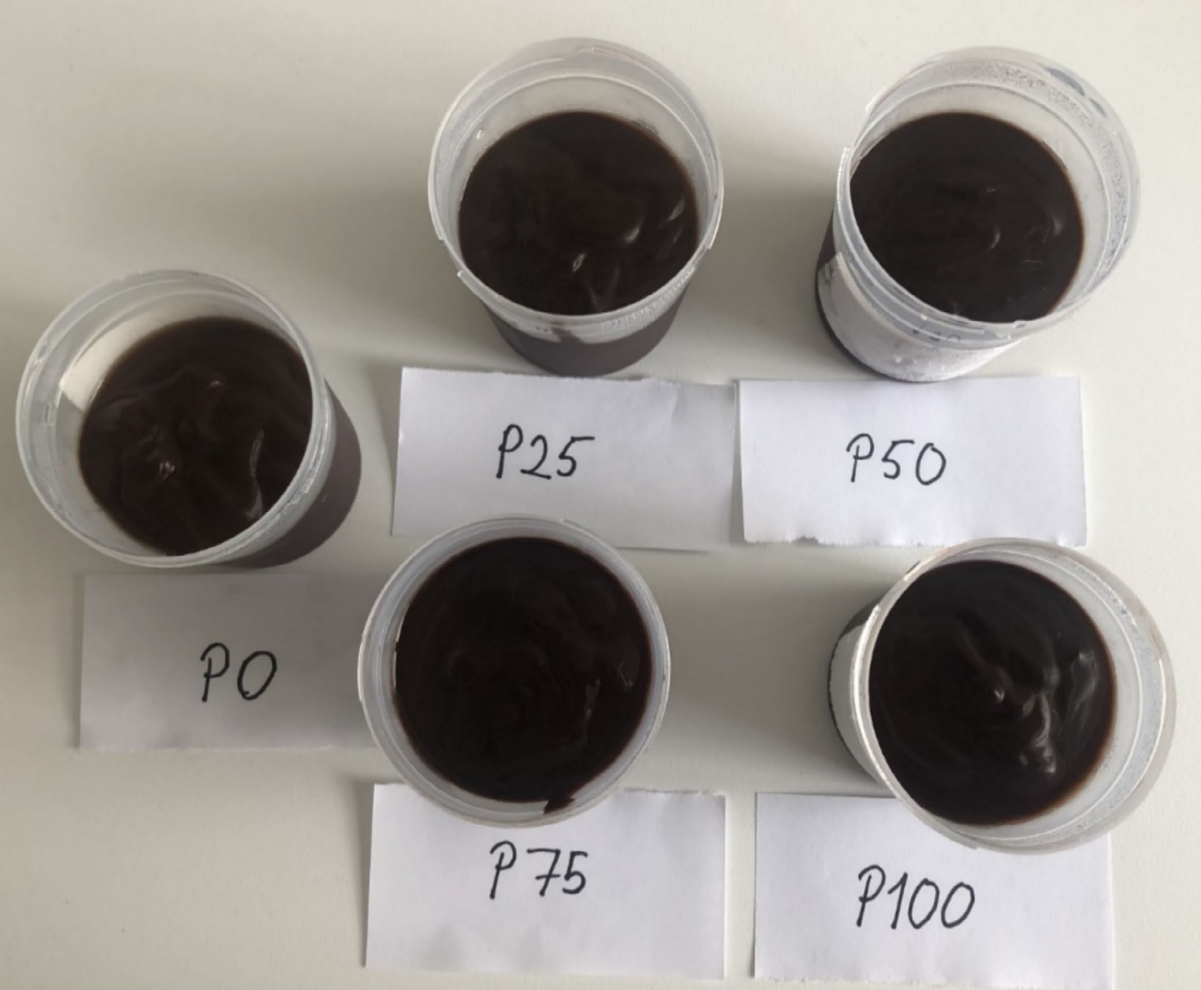
Pudding samples.

In the sensory analysis form given to the panelists, the phrases “brown or light brown” and “not acceptable color” were used to distinguish between desirable and undesirable color‐appearance characteristics, respectively. The panelists gave scores to the pudding samples from 1 to 9 based on their preferences. The color‐appearance scores of the puddings made from pumpkin seed milk were higher than the cow's milk pudding (P0‐control). The color‐appearance score increased significantly from 7.37 (=good) for P0 to 8.20 (=very good) for P100 (*p* < 0.05). The color‐appearance scores increased as the amount of pumpkin seed milk used in the pudding increased. The findings are consistent with the findings of Auti (2024), who found that the color‐appearance scores of milkshakes increased with the use of pumpkin seed powder; Asaduzzaman et al. (2025), who discovered that the color scores of cookies increased with the addition of pumpkin seeds; and Yeniçeri (2019), who discovered that the color scores of yogurts increased with the addition of pumpkin seeds. In contrast to our study, Karlı and Koç (2023) reported that cow's milk pudding was favored more than pumpkin seed milk pudding in terms of color‐appearance. In addition, Dabija et al. (2018) reported that the use of pumpkin seeds decreased the color values of yogurts. The ratio of pumpkin seeds or milk used in pudding production and the dilution ratio of pumpkin seeds to milk may have led to different results. Pigments in pumpkin seeds are transferred to pumpkin seed milk, resulting in a color change in the desserts produced from it, which was appreciated by the panelists. The use of pumpkin seed milk increases the greenness of the color. This may lead to lower sensory scores in cocoa‐free puddings. Since we use cocoa in the production of puddings, as seen in Figure 6, the color‐appearance of the samples did not suffer, on the contrary, both the color‐appearance and general acceptability scores were high.

In the sensory analysis form given to the panelists, the desired properties in terms of structure‐consistency were described as follows: “The cross‐section taken with a spoon should have a full consistency and smooth structure, feel homogeneous and full‐bodied when tasted.” The unwanted texture and consistency characteristics were stated as “having a creeping structure when taken into the mouth, excessively fluid”. Panelists made sensory evaluations according to these criteria. The structure‐consistency values of the pudding samples were found between 6.40 (for P75) and 7.30 (for P50). Although the use of pumpkin seed milk up to 50% increased the texture scores of the pudding, the texture scores decreased beyond this ratio. The difference between the structure‐consistency scores of all samples found to be statistically significant (*p* < 0.05). Auti (2024) reported that the body‐texture scores of milkshakes increased statistically significantly with the use of pumpkin seed powder. Yeniçeri (2019) reported that consistency scores (both spoon‐feed and mouth‐feed) increased as the proportion of pumpkin seeds used in yogurt production increased. Asaduzzaman et al. (2025) found that the texture scores of cookies decreased with the addition of 5%, 10%, and 20% pumpkin seeds. Ayah et al. (2022) reported that the texture scores of puddings produced from plant‐based milk were lower than those produced from cow's milk. The lower protein and dry matter content of pumpkin seed milk compared to cow's milk (Table 2) decreased the WHC, viscosity, and firmness values (Table 4) of pumpkin seed milk pudding, resulting in lower structure‐consistency scores. Soluble and insoluble fibers present in plants can affect the structure of plant‐based milks. The presence of insoluble particles can create various sensory problems (Yazıcı et al. 2023) and accordingly change the sensory properties of products produced from these milks.

The panelists scored the taste‐odor based on the sensory analysis form's defined desirable (unique taste and odor) and undesirable (extremely sweet or tasteless, burnt taste, bitter taste) attributes. Panelists gave the highest taste‐odor score for P25. P25 scored 7.74 (=good‐quite good), while P0 scored 7.27. Taste‐odor scores decreased when more than 25% pumpkin seed milk was used, and P100 (5.90 points) was the least liked sample. There was a significant difference between the control (P0) and only P100 (*p* < 0.05). The unique odor and “nutty” taste of pumpkin seeds (Auti 2024) are assumed to have affected the pudding samples negatively. Kuru and Tontul (2020) reported that pumpkin seed milk has the lowest flavor value among different plant milk types. Karlı and Koç (2023) found that pudding made with cow's milk had a higher taste‐odor score (4.47) than pudding made with pumpkin seed milk (3.37). Hassan et al. (2012) found a significant decrease in taste scores with the use of pumpkin seed milk in fermented beverages made from rice (*p* < 0.05). Similarly, Ayah et al. (2022) reported that the use of plant‐based milks decreased taste‐odor scores in pudding. A previous study reported that using up to 40% pumpkin seed milk increased the flavor scores of breads (Jasper et al. 2020). According to Asaduzzaman et al. (2025), adding 5%, 10%, and 20% pumpkin seeds to cookies decreased their taste scores. The results obtained in these studies are compatible with the outcomes of the sensory analysis.

As in the taste‐odor results, P25 had the highest general acceptability value (7.57 = good‐quite good). P25 was followed by P50 (7.23) and P0 (7.10). Although P100 had the lowest score, its average score of 6.24 out of 9 indicates that it has a certain level of acceptability (6 = somewhat good). The difference between the control (P0) and only P100 was statistically significant (*p* < 0.05). Pre‐treatments (soaking, roasting, cooking, etc.) applied to the raw materials used in the production of plant‐based milk affect the sensory acceptability and general appreciation values of the products. Consistent with our study, Karlı and Koç (2023) reported that the general acceptability values were lower in pumpkin seed milk pudding. In addition, Hassan et al. (2012) reported that the acceptance value of fermented beverages decreased with the inclusion of pumpkin seed milk. In a study, it was reported that pumpkin seed milk had the lowest acceptability value among different plant milk types (Kuru and Tontul 2020). Ayah et al. (2022) found that puddings made with plant‐based milk scored significantly lower overall than those made with cow milk. Jasper et al. (2020) reported that using up to 40% pumpkin seed milk increased the overall acceptability values of breads. Yeniçeri (2019) reported that general acceptability scores increased with the use of pumpkin seeds in yogurt production and with the increase in the usage rate. The fact that pumpkin seeds receive different sensory scores in different products depends on the usage rate and their compatibility with the product in which they are used.

## 4 Conclusion

The consumption of plant‐based milks has become more popular in recent years. Plant‐based milks, which are generally consumed directly, have limited use in the preparation of different foods. In this study, it was aimed to investigate the physicochemical, textural, rheological, antioxidant, and sensory properties of cocoa pudding made with different ratios of milk obtained from pumpkin seeds, which have nutritional and functional properties. The use of pumpkin seed milk in pudding production caused a decrease in dry matter, ash, protein, WHC, viscosity, firmness, adhesiveness, color (L*, a*, b*, C*), *G*′ and *G*″ values, and an increase in pH, antioxidant activity, total phenolic content, and color‐appearance scores. The highest antioxidant activity and total phenolic substance values in the P100 sample are important results for the study. Pudding produced with 100% pumpkin seed milk provided a 37.20% increase in antioxidant activity and a 19.50% increase in total phenolic content compared to the control pudding. As a result of sensory evaluation, P100, P50, and P25 received the highest scores for color‐appearance, structure‐consistency, both taste‐odor, and general acceptability, respectively. It was observed that using up to 50% pumpkin seed milk instead of cow's milk in pudding production did not cause a significant difference in WHC, firmness, adhesiveness, taste‐odor, and general acceptability criteria compared to the control (*p* > 0.05). Therefore, the use of up to 50% pumpkin seed milk in pudding production is acceptable. The use of pumpkin seed milk in pudding is valuable in terms of product development/diversity for both producers and consumers in these times of increasing interest in plant‐based products. Pudding made with pumpkin seed milk is an alternative product for individuals who prefer to consume plant‐based foods or who are forced to do so for health reasons. Pumpkin seed milk can be used alone or in combination with cow's milk to improve the nutritional, functional, and sensory properties of the pudding. Further research may focus on the shelf life of pumpkin seed milk pudding. In the production of pumpkin seed milk, undesirable tastes and odors originating from pumpkin seeds can be removed, and more sensory‐acceptable products can be produced.

## Author Contributions

**Başak Aygün:** conceptualization (supporting), investigation (equal), resources (equal), writing – original draft (equal). **Ahmet Emirmustafaoğlu:** data curation (supporting), formal analysis (supporting), methodology (lead), project administration (lead), supervision (supporting), validation (supporting), writing – review and editing (supporting).

## Ethics Statement

The study was approved by the Bolu Abant İzzet Baysal University Ethics Committee with registration number 2022/424.

## Conflicts of Interest

The authors declare no conflicts of interest.

## Data Availability

Data available on request from the authors.
